# IL-6 Contributes to the Defective Osteogenesis of Bone Marrow Stromal Cells from the Vertebral Body of the Glucocorticoid-Induced Osteoporotic Mouse

**DOI:** 10.1371/journal.pone.0154677

**Published:** 2016-04-29

**Authors:** Xiang Li, Zong-yu Zhou, Yuan-yuan Zhang, Hui-lin Yang

**Affiliations:** 1 Department of Orthopedics, the First Affiliated Hospital of Soochow University, Suzhou, 215006, People’s Republic of China; 2 Department of Orthopedics, Huai'an First People's Hospital, Huai'an, 223300, People’s Republic of China; 3 Department of Orthopedics, Huaiyin hospital of Huai’an city, Huai'an, 223300, People’s Republic of China; 4 Department of pediatrics, Huai'an First People's Hospital, Huai'an, 223300, People’s Republic of China; Faculté de médecine de Nantes, FRANCE

## Abstract

Osteoporosis is one of the most prevalent skeletal system diseases. It is characterized by a decrease in bone mass and microarchitectural changes in bone tissue that lead to an attenuation of bone resistance and susceptibility to fracture. Vertebral fracture is by far the most prevalent osteoporotic fracture. In the musculoskeletal system, osteoblasts, originated from bone marrow stromal cells (BMSC), are responsible for osteoid synthesis and mineralization. In osteoporosis, BMSC osteogenic differentiation is defective. However, to date, what leads to the defective BMSC osteogenesis in osteoporosis remains an open question. In the current study, we made attempts to answer this question. A mouse model of glucocorticoid-induced osteoporosis (GIO) was established and BMSC were isolated from vertebral body. The impairment of osteogenesis was observed in BMSC of osteoporotic vertebral body. The expression profiles of thirty-six factors, which play important roles in bone metabolisms, were compared through antibody array between normal and osteoporotic BMSC. Significantly higher secretion level of IL-6 was observed in osteoporotic BMSCs compared with normal control. We provided evidences that IL-6 over-secretion impaired osteogenesis of osteoporotic BMSC. Further, it was observed that β-catenin activity was inhibited in response to IL-6 over-secretion. More importantly, *in vivo* administration of IL-6 neutralizing antibody was found to be helpful to rescue the osteoporotic phenotype of mouse vertebral body. Our study provides a deeper insight into the pathophysiology of osteoporosis and identifies IL-6 as a promising target for osteoporosis therapy.

## Introduction

Osteoporosis is one of the most prevalent skeletal system diseases. It is characterized by a decrease in bone mass and microarchitectural changes in bone tissue that lead to an attenuation of bone resistance and susceptibility to fracture. Primary osteoporosis is associated with menopause and ageing. Secondary osteoporosis is always resulted from some metabolic diseases, lifestyle, genetic disorders and drug therapies. the adverse effects of glucocorticoid overdose on bone have been revealed for more than 80 years [1], but the precise cellular and molecular basis remains largely unknown. Today, glucocorticoid-induced osteoporosis (GIO) is now third in frequency following postmenopausal and senile osteoporosis.

Bone loss in response to glucocorticoid overdose affects both cortical and cancellous bone and has a predilection for the axial skeleton. Therefore, spontaneous vertebrae fractures are often present in the disorder [2, 3]. Osteoporotic vertebral fracture (OVF) is by far the most prevalent osteoporotic fracture. In addition to pain, osteoporotic vertebral fractures result in immobility that can lead to chest infection, muscle loss, the inability to cope with daily activities, and social isolation [4].

One of key features of GIO is decreased bone formation [5]. However, the mechanisms underlying this remain elusive. Decreased bone formation and *in situ* death of isolated segments of the proximal femur reveal that glucocorticoid overdose may decrease the osteoblast production [3]. In the musculoskeletal system, osteoblasts are originated from bone marrow stromal cells (BMSCs). Therefore, BMSC is a promising target for elucidating the pathophysiological mechanisms of vertebral osteoporosis and developing effective methods to treat OVF. Some previous reports have demonstrated that BMSC osteogenesis is defective in osteoporosis [6, 7]. Enhancing BMSC osteogenesis will contribute to the increase in bone mass of osteoporotic bone. However, to date, the cause of the impairment of BMSC osteogenesis in osteoporosis remains an open question.

Bone marrow represents a complicated microenvironment. The multiple kinds of cells in bone marrow interact intensely through locally produced factors, the extracellular matrix components, and systemic factors [8, 9] in autocrine, paracrine and endocrine modes. BMSCs’ commitment towards the osteoblast requires suitable initiation factors in the bone marrow to activate lineage-specific transcriptional factors. In osteoporosis, distinctive bone marrow conditions provide support for the development and maintenance of unbalanced bone formation and resorption [10, 11]. In this sense, elucidating the abnormal changes in osteoporotic bone marrow microenvironments will facility our understanding of the cause of the impairment of BMSC osteogenesis in osteoporosis and our efforts to enhance BMSC osteogenesis in osteoporosis.

Interleukin (IL)-6 is involved in a spectrum of age-associated diseases, such as osteoporosis whose initiation and time course is affected by proinflammatory cytokines. Enhancement of IL-6 level is observed in the ongoing processes of aging and menopause which is manifested by osteoclast activation and bone resorption [12, 13]. Clinically, enhanced IL-6 production is reported to be associated with osteoporosis [14, 15]. Recently, increased IL-6 soluble receptors have been reported to be a predictive vane in evaluating hip fracture risks [16], and there is a significant correlation between serum levels of IL-6 and CRP and BMD [17]. However, the role of IL-6 in GIO vertebral fracture and the underlying molecular mechanisms remain unknown.

In the current study, we made attempts to elucidate the molecular mechanisms underlying the defective BMSC osteogenesis in GIO. A GIO mouse model was established and BMSCs were isolated from vertebral body. The defective osteogenesis was observed in BMSCs of osteoporotic vertebral body. The expression profiles of thirty-six factors, which play important roles in bone metabolisms, were compared through antibody array between normal and osteoporotic BMSCs. Significantly higher secretion level of IL-6 was observed in osteoporotic BMSCs compared with normal control. We provided evidences that IL-6 over-secretion impaired osteogenesis of osteoporotic BMSCs. Further, it was observed that β-catenin activity was inhibited in response to IL-6 over-secretion. More importantly, *in vivo* administration of IL-6 neutralizing antibody was found to be helpful to rescue the osteoporotic phenotype of mouse vertebral body.

## Materials and Methods

### Animals

Four-month-old male Swiss Webster mice were housed under standard laboratory conditions. Global and spinal BMD scans were performed using dual-energy x-ray absorptiometry on a QDR-2000 Plus densitometer (Hologic, Waltham, MA) at week 0 and 4 after pellet implantation. The percentage of BMD change ((BMD_**w4**_-BMD_**w0**_)/BMD_**w0**_) was calculated. The ethics committee of animal welfare and use in medical college of Soochow University approved the protocols of animal experiments.

Slow-release pellets (Innovative Research of America, Sarasota, FL) containing placebo or 2.1 mg/kg/d of prednisolone were implanted subcutaneously for 4 weeks. There were 10 animals per group. At the time the mice were sacrificed, the thoracic and lumbar vertebrae (L1–L4) were prepared for BMSC isolation; L5 was used for histomorphometric analysis. Serum specimens were also taken for the measurement of osteocalcin and C-telopeptide of type I collagen (CTX-I) (Biomedical Technologies, Stoughton, MA).

The male 10-week-old IL-6 KO mice (B6.129S2-Il6^tm1Kopf^/J, Jackson Lab) and the littermates control mice on a C57BL/6J background, housed in the same facility, were used. There were 10 animals per group. The primers used in genotyping were: Common: 5’-TTCCATCCAGTTGCCTTCTTGG-3’, Wild type reverse: 5’-TTCTCATTTCCACGATTTCCCAG-3’, Mutant reverse: 5’-CCGGAGAACCTGCGTGCAATCC-3’. The PCR products were separated by gel electrophoresis on a 1.5% agarose gel. The mutant band was 380bp and the wild type band was 174bp.

### Bone histomorphometric analysis

The L5 lumbar vertebrae were fixed with 10% formalin in phosphate-buffered saline (pH 7.4), and embedded undecalcified in methyl methacrylate. The histomorphometric examination was performed using a computer and digitizer tablet (OsteoMetrics Inc., Atlanta, GA) interfaced to a Zeiss Axioscope (Carl Zeiss, Inc., Thornwood, NY) with a drawing tube attachment. The cancellous measurements were two-dimensional, confined to the secondary spongiosa, and made at a magnification of 400. The region of interest was selected by a boundary beginning 0.5 mm proximal to the midpoint of the growth plate, non-inclusive of cortical bone, and extending proximally for a total area of approximately 2.8 mm^2^. The terminology and units used are those recommended by the Histomorphometry Nomenclature Committee of the American Society for Bone and Mineral Research [18].

### Histology

L4 vertebrae was fixed in 10% formalin, decalcified, and embedded in paraffin. Serial sagittal sections were cut every 10 μm. The sections were stained with hematoxylin & orange G (H&OG).

### Cell culture

The BMSCs were flushed out of the vertebrae with αMEM and cultured in αMEM containing 20% fetal bovine serum (FBS) and 1% penicillin–streptomycin (all from Hyclone, Logan, UT, USA) at 37°C and 5% CO_2_. The non-adherent cells were removed by replacing the medium after 3 days.

BMSCs were washed by PBS and stained with the respective antibodies including anti-mouse FITC-CD90, FITC-CD105 and FITC-CD45 (BD Bioscience) at 4°C for 30 min. The mouse FITC-IgG was used as the isotype control. The cell pellets were resuspended in PBS and analyzed with flow cytometry (Calibur, Becton Dickinson, USA).

IWR-1 (10^−6^ M) (Sigma, St. Louis, MO, USA) was used to block Wnt/β-catenin signaling pathway. LiCl (20 mM) (Sigma) was used to activate Wnt/β-catenin signaling pathway. AG490 (50 μM) (Sigma) was used to block STAT3 signaling.

To induce osteogenesis, the BMSCs were treated with BMP-2 (R&D Systems, Minneapolis, MN, USA) at a final concentration of 100 ng/ml.

### MTT

BMSCs were plated into 96-well plates at a density of 7000 cells/well. On day 1 to day 7 after plating, 10 μL 5mg/mL MTT (Sigma, USA) was added into the culture medium for 4 h. The absorbance was read at 490nm by microplate reader. Each sample was analyzed in triplicate.

### ALP staining and quantification

BMSCs induced to osteogenic differentiation were washed with PBS and fixed with 4% paraformaldehyde for 10 min at 4°C. Then the cells were incubated in 0.1% naphthol AS-MX phosphate (Sigma) and 0.1% fast red violet LB salt (Sigma) in 2-amino-2-methyl-1,3-propanediol (Sigma) for 10 min at room temperature, washed with PBS, and then observed under a digital camera.

For ALP quantification, BMSCs induced to osteogenic differentiation were washed with PBS and then scraped into ddH2O. Three cycles of freezing and thawing were performed. ALP activity was determined at 405 nm using p-nitrophenyl phosphate (pNPP) (Sigma, St. Louis, MO) as the substrate. Total protein content was determined with the BCA method, read at 562 nm and calculated according to a series of albumin (BSA) standards. ALP levels were normalized to the total protein content at the end of the experiment. All experiments were conducted in triplicate.

### Alizarin Red S staining and quantification

Osteoblast maturation was evaluated by mineralized nodules staining with Alizarin Red. After fixation, the cells were washed with PBS and incubated in 40 mM Alizarin Red (pH 4.2) for 30 min at 37°C, then washed with PBS and imaged.

Decalcification was performed using 0.1 M HCl overnight at 4°C. Then, 20 μL of samples were transferred to the test tubes containing 1 mL of methyl thymol blue solution and 1 mL of alkaline solution. Absorbance was determined at 610 nm.

### Quantification of osteoblasts in *ex vivo* bone marrow cultures

The number of CFU-osteoblasts (CFU-OB) in the bone marrow preparations was determined as described previously [19]. Briefly, cells were seeded at a density of 3×10^6^ cells/10 cm^2^ well and maintained for 25 days in αMEM containing 15% FBS, 50 mM ascorbic acid, and 10 mM β-glycerophosphate (Sigma Chemical Co., St. Louis, MO) with one-half of the medium replaced every 5 d. After fixation in 50% ethanol and 18% formaldehyde, the cultures were stained using Von Kossa’s method to visualize and quantify the number of colonies containing mineralized bone matrix.

### Reverse transcription (RT)-PCR and real-time PCR

Total RNA was isolated from BMSCs using an RNeasy Mini Kit (Qiagen, Valencia, CA, USA). For RT-PCR, single-stranded cDNA was reverse-transcribed from 1 μg of total RNA using an oligo-dT primer. PCR was performed using 1 μl of cDNA and the following cycling parameters: 30 cycles of 94°C for 40 s, 60°C for 40 s, and 72°C for 40 s. The PCR products were then analyzed by agarose gel electrophoresis. Quantitative PCR was performed using the ABI Prism 7500 (Applied BioSystems, Foster City, CA, USA) and the SYBR Green PCR Master Mix (Takara Bio Inc., Otsu, Japan). The cycling conditions were as follows: 94°C for 30 s followed by 40 cycles of 94°C for 5 s and 60°C for 34 s. The comparative 2^−ΔΔCt^ method was used to calculate the relative expression of each target gene. β-actin was used as an internal control. The primer sequences are listed in S1 Table.

### Western blot

Cells were lysed on ice for 30 min in a buffer containing 50 mM Tris-HCl, pH 7.4, 150 mM NaCl, 1% Nonidet P-40, and 0.1% SDS supplemented with protease inhibitors (10 g/ml leupeptin, 10 g/ml pepstatin A, and 10 g/ml aprotinin). The proteins were separated by SDS-PAGE, transferred to a nitrocellulose membrane, and detected using anti-IL-6 (#12912, Cell Signaling Technology, Danvers, MA, USA), anti-p-STAT3 (Y705) (#9145, CST), anti-STAT3 (#9139, CST), anti-non-phospho (active) β-catenin (#19807, CST), and anti-β-actin (#3700, CST) antibodies. The proteins were visualized using an enhanced chemiluminescence system (GE Healthcare, Piscataway, NJ, USA).

### Antibody arrays

Semi-quantitative sandwich-based antibody arrays (RayBio^®^ Human Cytokine Array L-Series) were employed to detect 36 markers of bone metabolism on a glass slide matrix. The detection antibodies were biotin-labeled and combined to generate a single cocktail reagent for later use. The printed slides were placed in chamber assemblies so that each array could be incubated with a different sample. After blocking with a blocking buffer, the arrays were incubated with the whole-cell lysis samples. Following extensive washing to remove non-specific binding, the cocktail of biotinylated detection antibodies was added to the arrays. After extensive washing, the array slides were incubated with a streptavidin-conjugated fluor (HiLyte Fluor^™^ 532 from Anaspec, Fremont, CA). The fluorescent signals were then visualized in the green channel using a laser-based scanner system (GenePix 4200A, Molecular Dynamics, Sunnyvale, CA).

### *In vitro* and *In vivo* neutralization of IL-6

To block the IL-6 activity, IL-6 neutralization antibody (#500-P56, Peprotech, Rocky Hill, NJ, USA) was added to the culture medium of BMSCs at a concentration of 0.8 μg/ml. IgG was used as control.

Mice implanted with the placebo or prednisolone pellets were intraperitoneally injected with 500μg of a neutralizing IL-6 antibody (#BE0046, BioXcell) or an IgG isotype control (#BE0088, BioXcell) 4, 11, 18, and 25 days after pellet implantation. The mice were sacrificed on day 28.

### RNAi

Lentiviral vectors loading shRNAs against IL-6 were purchased from Genecopoeia^®^ (Rockville, MD, USA). 293T cells were plated in a 10 cm dish and the transfection mixture was added directly to the culture medium at 70–80% confluence. Following transfection, the samples were incubated in a CO_2_ incubator at 37°C for 48 h and the virus particle-containing medium was then collected. Lentivirus titer was measured by Lenti-X p24 Rapid titer kit (Clontech, Mountain View, CA, USA). After infection of BMSC by lentivirus loading shRNAs against IL-6 (MOI = 25), RT-PCR and western blot were employed to analyze the IL-6 expression.

### Statistical analysis

Statistical significance was calculated by Student’s t-test for two-sample comparisons and one-way ANOVA for multiple comparisons using the software SPSS 16.0. Tukey’s test was used to identify significant differences in ANOVA. Statistical significance was determined using data from at least three independent experiments. *p* values<0.05 were defined as significant. All of the data are presented as the mean±SD unless otherwise specified.

## Results

### BMSC osteoporosis is defective in the vertebral body of the osteoporotic mouse

In the current study, a GIO mouse model was established. In the prednisolone group, the global and spinal BMDs at day 28 were significantly lower than in the placebo group (Table 1). The level of serum osteocalcin, a marker of osteoblast activity, was decreased by approximately 50% in the prednisolone group when compared with the placebo group (Table 1). The level of serum CTX-I, a marker of bone resorption, was increased by over 30% in the prednisolone group when compared with the placebo group (Table 1).

**Table 1:** BMD and serum biochemical measurements in mice with and without 4-week prednisolone administration. Data shown are the mean±SD. There are ten animals per group.

Parameters	Placebo	Prednisolone
Global BMD (% change)	-3.2±1.6	-7.8±2.1 *
Spinal BMD (% change)	-4.3±2.2	-9.1±3.7 *
Osteocalcin (mg/L)	82.7±8.6	43.2±6.5 **
CTX-I (ng/mL)	8.4±1.5	11.2±1.4*

*P<0.05 *vs*. placebo;

**P<0.01 *vs*. placebo.

Consistent with the BMD results, there was a significant decline in the vertebral cancellous bone area, trabecular width, and trabecular number and a significant increase in the trabecular spacing in the prednisolone group when compared with the placebo group (Table 2). A significant decrease in osteoblast surface (% bone surface) and increase in osteoclast surface (% bone surface) were observed in the prednisolone group when compared with the placebo group (Table 2). The data described above suggested that there were excessive bone resorption and defective bone formation in response to prednisolone treatment.

**Table 2:** Vertebral cancellous bone histomorphometry analysis in mice with and without 4-week prednisolone administration. ObS: Osteoblast surface, OcS: Osteoclast surface, BS: bone surface. Data shown are the mean±SD. There are ten animals per group.

Histomorphometric parameters	Placebo	Prednisolone
Bone area/tissue area (%)	14.5±2.1	4.9±1.4**
Trabecular thickness (μm)	41.6±2.6	28.3±4.2**
Trabecular spacing (μm)	454±71	597±42*
Trabecular number (mm^-1^)	1.82±0.36	1.32±0.22*
ObS/BS (%)	37.23±6.35	25.73±5.17*
OcS/BS (%)	2.25±0.47	4.59±0.72*

*P<0.05 *vs*. placebo;

**P<0.01 *vs*. placebo.

**Fig 1:**
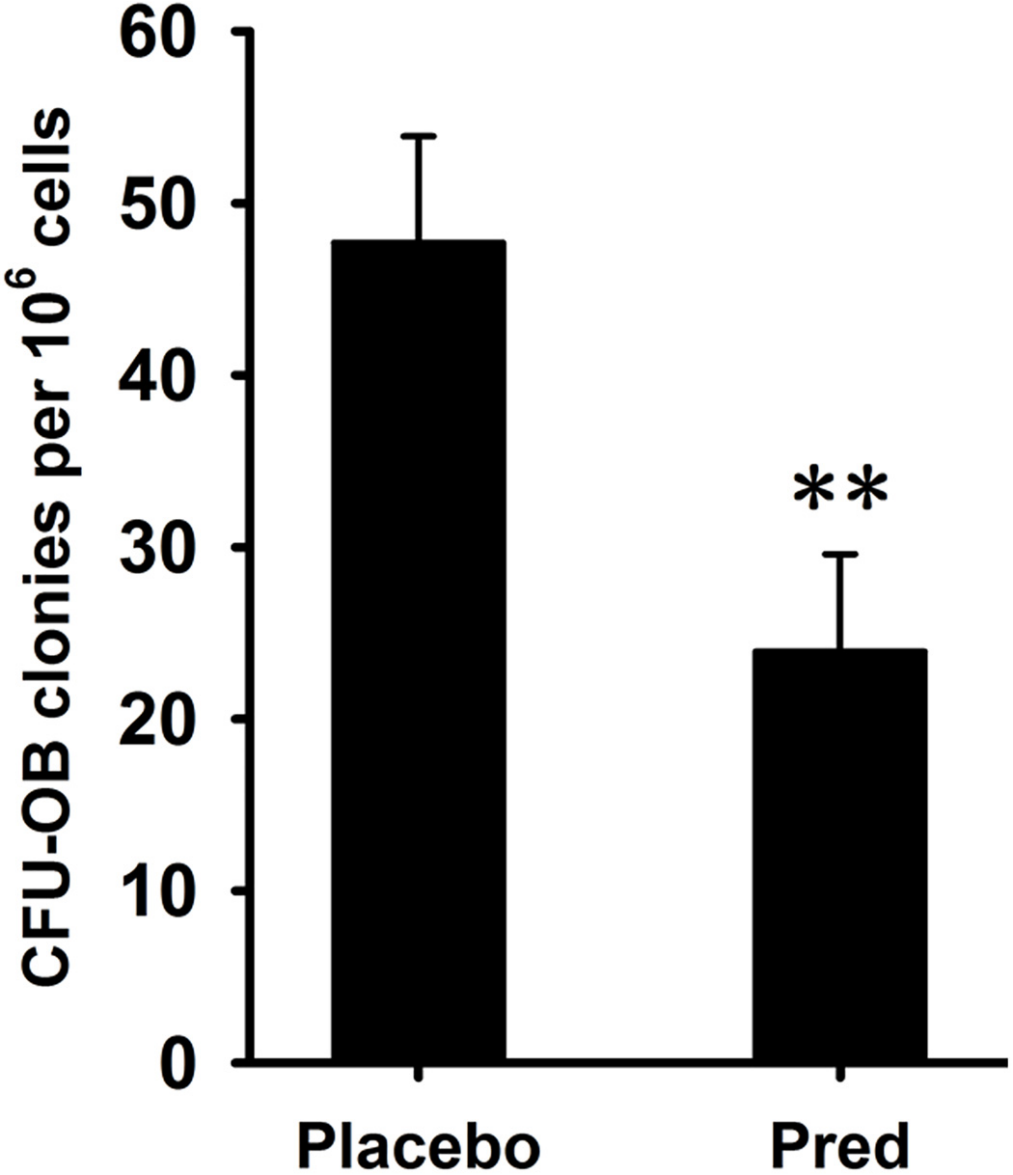
Quantification of CFU-OB. A glucocorticoid-induced osteoporosis (GIO) mouse model was established by subcutaneously implanting placebo- or prednisolone-releasing pellets into mice for 4 weeks. BMSCs were isolated from the vertebral body and cultured *in vitro*. The number of CFU-OB was quantified. The results are expressed as the number of CFU-OB per 10^6^ cells. The data are shown as the mean±SD. **: p<0.01.

**Fig 2:**
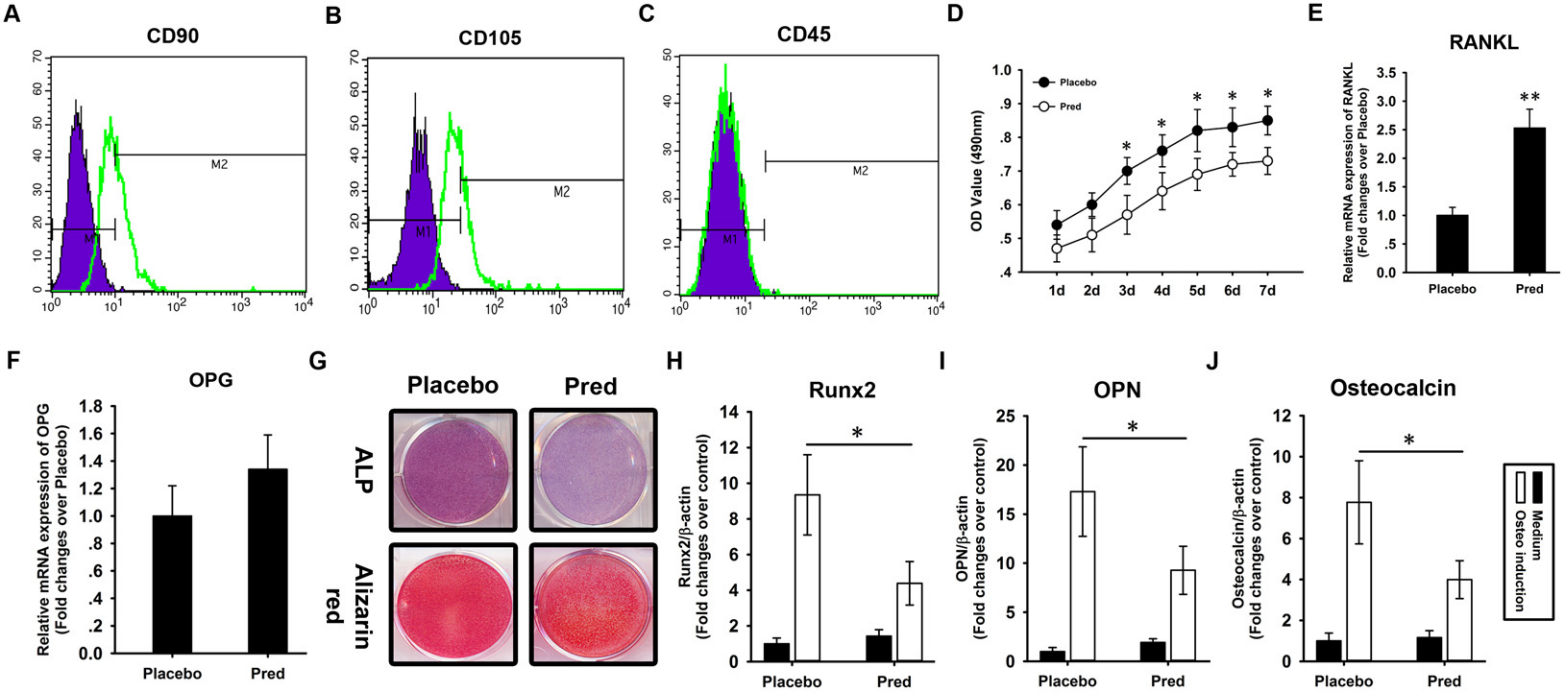
BMSC osteogenesis is defective in the vertebral body of the osteoporotic mouse. BMSCs were isolated from the vertebral bodies of mice treated with the placebo or prednisolone pellets, cultured *in vitro*, and analyzed by flow cytometry to detect the expression of the mesenchymal surface markers CD90 **(A)** and CD105 **(B)** and the hematopoietic surface marker CD45 **(C)**. MTT assay was used to analyze the proliferation of BMSCs from prednisolone and placebo group **(D)**. RANKL **(E)** and OPG **(F)** mRNA expression were analyzed using quantitative RT-PCR. The BMSCs were then induced to undergo osteogenesis. Alkaline phosphatase (ALP) and Alizarin Red staining were performed at weeks 1 and 3, respectively **(G)**. At week 2, total RNA was extracted and subjected to quantitative RT-PCR analysis using primers for Runx2 **(H)**, OPN **(I),** and OC **(J)**. β-actin was used as an internal control. The results are expressed as the fold-change relative to normal BMSCs without induction. The data are shown as the mean±SD. *: p<0.05. **: p<0.01.

In BMSC cultures from the vertebral bodies of the prednisolone group, there were 50% fewer CFU-OBs than in the placebo group (Fig 1A). Flow cytometry revealed that these cells were positive for the mesenchymal surface markers CD90 (Fig 2A) and CD105 (Fig 2B) but negative for the hematopoietic surface marker CD45 (Fig 2C). BMSC proliferation was analyzed by MTT assay. During a 7-day culture period, significant decrease in the proliferation rate was observed from day 3 to 7 in BMSCs from the prednisolone group compared with the placebo group (Fig 2D). RANKL and OPG mRNA expression in BMSCs was analyzed using quantitative RT-PCR. Significant upregulation of RANKL mRNA expression was observed in BMSCs from the prednisolone group compared with the placebo group (Fig 2E). There was no difference in OPG mRNA expression in BMSCs between the prednisolone and placebo group (Fig 2F).

The BMSCs were induced to undergo osteogenesis *in vitro*. At week 1, ALP staining revealed that ALP activation was weaker in the BMSCs from the prednisolone group compared with those from the placebo group (Fig 2G). At week 3, Alizarin Red staining revealed the same pattern as the ALP staining (Fig 2G). Total RNA was extracted at week 2 and subjected to quantitative RT-PCR to determine the expression of several osteogenic markers including Runx2, OPN, and OC. The qRT-PCR revealed that the prednisolone group showed a less significant BMP2-induced activation of Runx2 (Fig 2H) and lower levels of OPN (Fig 2I) and OC (Fig 2J) mRNA than the placebo group.

### IL-6-STAT3 signaling is over-activated in osteoporotic BMSCs

**Fig 3:**
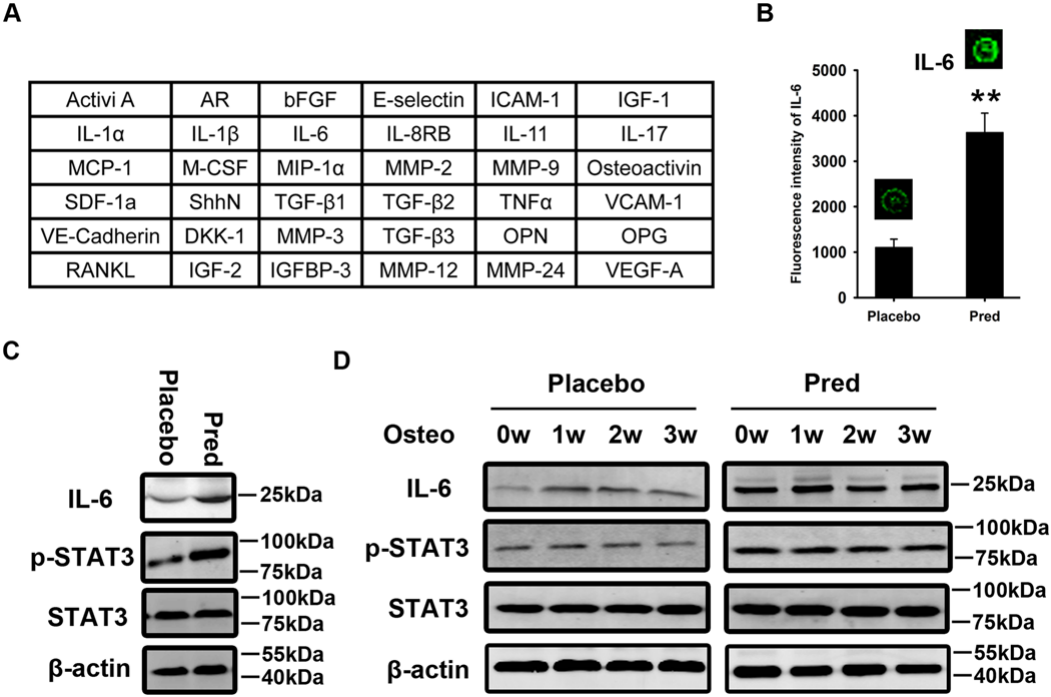
IL-6-STAT3 signaling is over-activated in osteoporotic BMSCs. The expression of thirty-six important bone metabolism markers was measured in normal and osteoporotic BMSCs treated with BMP2 for 7 days using an antibody array **(A)**. The fluorescence intensity represents the relative expression level of the indicated protein. Of the thirty-six bone metabolism markers investigated, interleukin-6 (IL-6) was found to be upregulated in osteoporotic BMSCs compared with normal BMSCs **(B)**. The data are shown as the mean±SD. Total protein was harvested from the normal and osteoporotic BMSCs and subjected to western blotting using IL-6, p-STAT3, and STAT3 antibodies **(C)**. Normal and osteoporotic BMSCs were induced to undergo osteogenesis. Zero, 1, 2, and 3 weeks after induction, total protein was harvested for western blotting using IL-6, p-STAT3, and STAT3 antibodies **(D)**. β-actin was used as an internal control. **: p<0.01, Pred *vs*. Placebo.

Having observed the defective osteogenesis in osteoporotic BMSCs, we aimed to elucidate the underlying molecular mechanisms. Total protein was harvested from normal and osteoporotic BMSCs treated with BMP2 for 7 days. The protein expression levels of thirty-six bone metabolism markers were assessed using an antibody array (Fig 3A). The fluorescence intensity represented the relative expression level of the indicated protein. Interleukin-6 (IL-6) was upregulated 3-fold in osteoporotic BMSCs compared with the normal control (Fig 3B). Western blotting confirmed the upregulation of IL-6 and revealed a significant increase in the phosphorylation of signal transducer and activator of transcription 3 (STAT3), downstream factor of IL-6, in osteoporotic BMSCs compared with normal BMSCs (Fig 3C). It should be noted that in addition to IL-6, some other factors secreted by BMSCs, including G-CSF, leptin, oncostatin M and IL-11 could also activate STAT3. The expression levels of these factors were measured. There was mild downregulation of G-CSF expression in BMSCs from prednisolone group compared with placebo (S1 Fig). And no differences in leptin, oncostatin M and IL-11 expressions were observed in BMSCs between placebo and prednisolone group (S1 Fig).

BMSCs were induced to undergo osteogenesis *in vitro*. Zero, 1, 2, and 3 weeks after induction, total protein was harvested from the normal and osteoporotic BMSCs. Western blotting revealed a significant upregulation of IL-6 and activation of STAT3 in the osteoporotic BMSCs during *in vitro* osteogenesis when compared with the normal BMSCs (Fig 3D).

### IL-6 contributes to the defective osteogenesis of osteoporotic BMSCs

**Fig 4:**
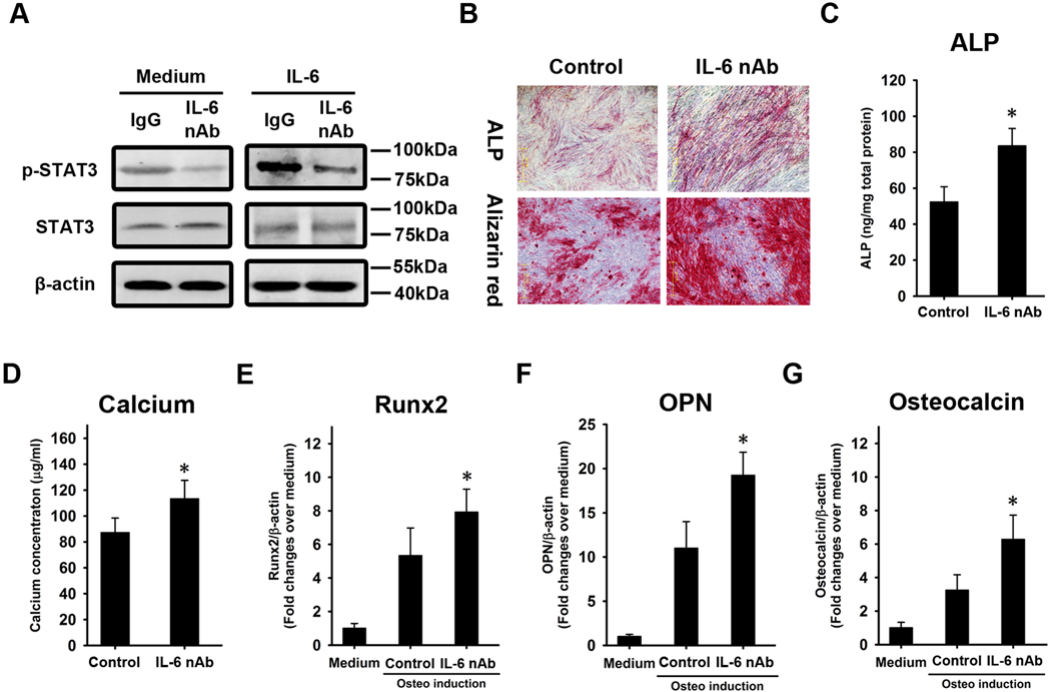
An IL-6 neutralizing antibody enhances the osteogenesis of osteoporotic BMSCs. Osteoporotic BMSCs, with and without IL-6 treatment, were treated by an IL-6 neutralizing antibody. IgG was used as a control. STAT3 phosphorylation was analyzed by western blot **(A)**. Alkaline phosphatase (ALP) and Alizarin Red staining were performed at weeks 1 and 3, respectively **(B)**. The quantification of ALP **(C)** and measurement of calcium concentration **(D)** were performed. At week 2, total RNA was extracted and subjected to quantitative RT-PCR analysis using primers for Runx2 **(E)**, OPN **(F),** and OC **(G)**. β-actin was used as an internal control. The results are expressed as the fold-change relative to BMSCs without induction. The data are shown as the mean±SD. *: p<0.05, IL-6 nAb *vs*. control.

**Fig 5:**
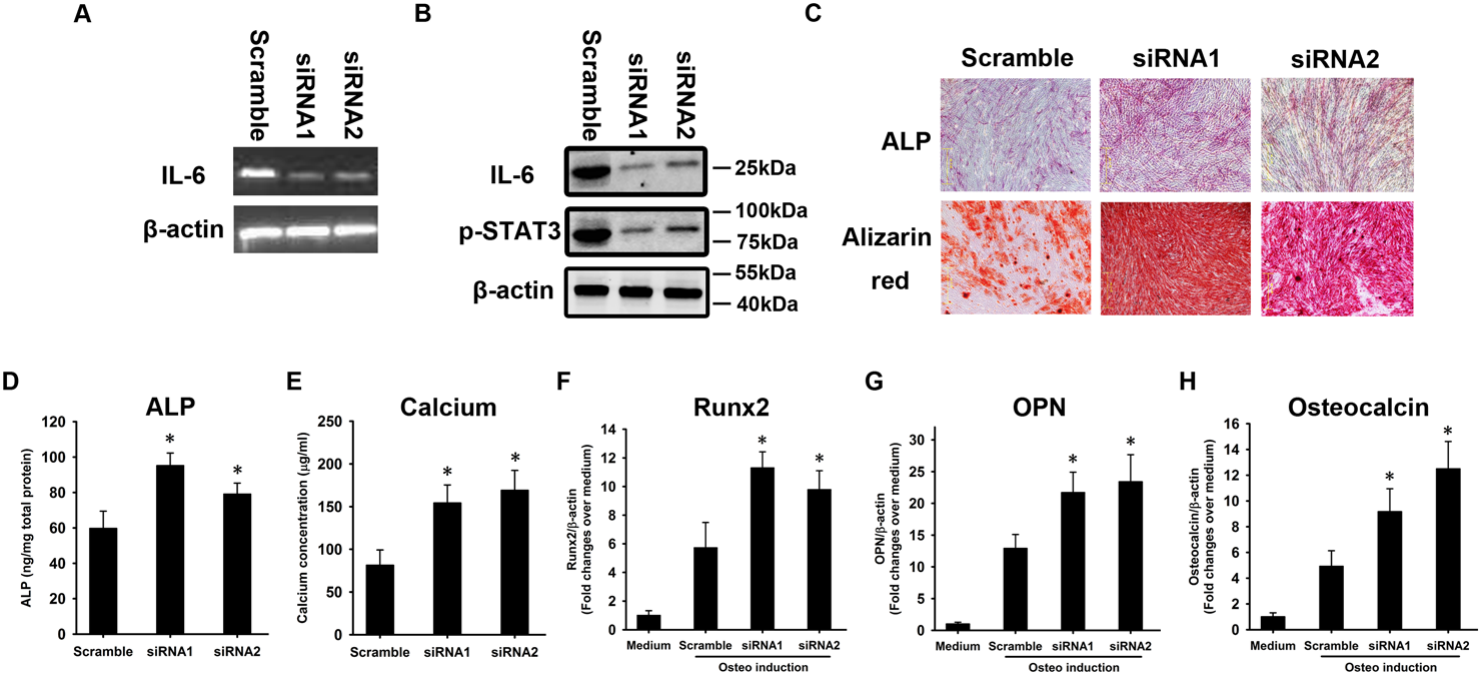
shRNAs against IL-6 enhance the osteogenesis of osteoporotic BMSCs. shRNAs were used to silence IL-6 expression. Silencing was confirmed by RT-PCR **(A)** and western blotting **(B)**. A scrambled sequence was used as a control. Alkaline phosphatase (ALP) and Alizarin Red staining were performed at weeks 1 and 3, respectively **(C)**. The quantification of ALP **(D)** and measurement of calcium concentration **(E)** were performed. At week 2, total RNA was extracted and subjected to quantitative RT-PCR using primers for Runx2 **(F)**, OPN **(G),** and OC **(H)**. β-actin was used as an internal control. The results are expressed as the fold-change relative to BMSCs without induction. The data are shown as the mean±SD. *: p<0.05, shRNA *vs*. scrambled control.

We asked whether the over-secretion of IL-6 was involved in the defective osteogenesis of osteoporotic BMSCs. To answer this question, an IL-6 neutralizing antibody was employed to block IL-6 signaling in osteoporotic BMSCs. Non-specific IgG was used as a control. STAT3 phosphorylation was downregulated in response to IL-6 neutralizing antibody (Fig 4A). The cells were then induced to undergo osteogenesis. At week 1, ALP staining revealed that ALP activation was stronger in response to the IL-6 neutralizing antibody when compared with the IgG control (Fig 4B). At week 3, Alizarin Red staining revealed the same pattern as the ALP staining (Fig 4B). The quantification of ALP (Fig 4C) and calcium concentration (Fig 4D) supported the staining data. Total RNA was extracted at week 2 and subjected to quantitative RT-PCR to determine the expression of Runx2, OPN, and OC. The qRT-PCR revealed a more significant BMP2-induced activation of Runx2 (Fig 4E) and higher levels of OPN (Fig 4F) and OC (Fig 4G) mRNA in response to the IL-6 neutralizing antibody compared with the IgG control. We used shRNA to silence IL-6 expression in osteoporotic BMSCs (Fig 5A and 5B). STAT3 was significantly inhibited in response to IL-6 knockdown (Fig 5B). The cells were then induced to undergo osteogenesis. At week 1, ALP staining revealed that ALP activation was stronger in response to IL-6 knockdown when compared to treatment with a scrambled control (Fig 5C). At week 3, Alizarin Red staining revealed the same pattern as the ALP staining (Fig 5C). The quantification of ALP (Fig 5D) and calcium concentration (Fig 5E) supported the staining data. Total RNA was extracted at week 2 and subjected to quantitative RT-PCR to determine the expression of Runx2, OPN, and OC. The qRT-PCR revealed a more significant BMP2-induced activation of Runx2 (Fig 5F) and higher levels of OPN (Fig 5G) and OC (Fig 5H) mRNA in response to IL-6 knockdown compared with the scrambled control.

### Over-activation of IL-6-STAT3 signaling leads to the inhibition of β-catenin activity

**Fig 6:**
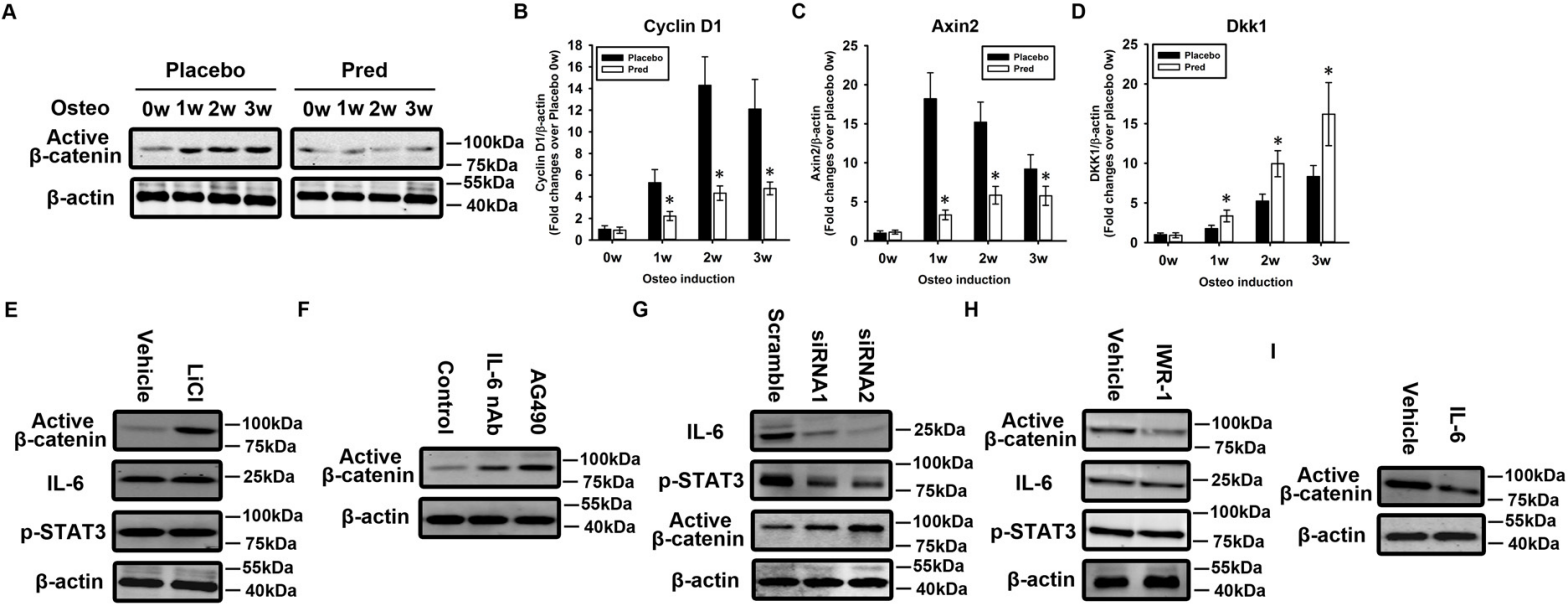
Over-activation of IL-6-STAT3 signaling inhibits β-catenin activity. Normal and osteoporotic BMSCs were induced to undergo osteogenesis. Zero, 1, 2, and 3 weeks after induction, total protein was harvested, and western blotting was carried out using an antibody against non-phospho (active) β-catenin **(A)**. Total RNA was harvested, and quantitative RT-PCR was carried out using primers for cyclin D1 **(B)**, Axin2 **(C),** and Dkk1 **(D)**. The results are expressed as the fold-change relative to normal BMSCs without induction. The data are shown as the mean±SD. Osteoporotic BMSCs were treated with LiCl to activate β-catenin **(E)**. An IL-6 neutralizing antibody and AG490 were used to block IL-6-STAT3 signaling **(F)**. shRNAs were used to silence IL-6 expression **(G)**. Normal BMSCs were treated with IWR-1 to inhibit β-catenin **(H)**. Total protein was harvested, and western blotting was carried out using antibodies against active β-catenin, IL-6, and pSTAT3. IL-6 was added to the medium **(I)**. Western blotting was performed using an antibody against active β-catenin. β-actin was used as an internal control. *: p<0.05, Pred *vs*. scrambled control.

We next attempted to elucidate the mechanisms by which IL-6 led to the defective osteogenesis of osteoporotic BMSCs. Wnt/β-catenin signaling plays an essential role in the osteogenic differentiation of BMSCs [20]. Therefore, we wondered whether there was crosstalk between IL-6 and β-catenin signaling. BMSCs were induced to undergo osteogenesis *in vitro*. Zero, 1, 2, and 3 weeks after induction, total protein and RNA were harvested from the normal and osteoporotic BMSCs. Western blotting revealed a significant downregulation of non-phospho (active) β-catenin in osteoporotic BMSCs during *in vitro* osteogenesis compared with normal BMSCs (Fig 6A). Accordingly, qRT-PCR revealed a significant downregulation in the mRNA expression of β-catenin target genes including cyclin D1 (Fig 6B), Axin2 (Fig 6C), and a significant upregulation of Wnt/β-catenin signaling inhibitor Dkk1 (Fig 6D) in osteoporotic BMSCs during *in vitro* osteogenesis compared with normal controls.

Osteoporotic BMSCs were treated with LiCl to activate wnt/β-catenin signaling. Western blotting confirmed the activation of β-catenin in response to LiCl treatment (Fig 6E). However, there was no change in IL-6 expression or STAT3 activation (Fig 6E), indicating that β-catenin does not function upstream of IL-6. In contrast, a significant upregulation of β-catenin activity was observed in osteoporotic BMSCs in response to an IL-6 neutralizing antibody and treatment with the STAT3 inhibitor AG490 (Fig 6F), suggesting that IL-6 acts upstream of β-catenin to negatively modulate β-catenin.

Since AG490 is not entirely STAT3 specific [21], shRNAs against IL-6 were used to silence IL-6 expression in osteoporotic BMSCs to block IL-6/STAT3 signaling specifically. Western blotting revealed that IL-6 and phospho-STAT3 were downregulated in response to IL-6 knockdown (Fig 6G). A significant upregulation of β-catenin activity was observed in response to the IL-6 shRNAs (Fig 6G), suggesting that there is a negative relationship between IL-6 and β-catenin.

Normal BMSCs were treated with IWR-1 to inhibit wnt/β-catenin signaling. Western blotting confirmed the inactivation of β-catenin in response to IWR-1 treatment (Fig 6H). However, there was no change in IL-6 expression or STAT3 activation (Fig 6H), indicating that β-catenin does not function upstream of IL-6. In contrast, a significant downregulation of β-catenin activity was observed in normal BMSCs following IL-6 treatment (Fig 6I), suggesting that IL-6 acts upstream of β-catenin to negatively modulate β-catenin.

**Fig 7:**
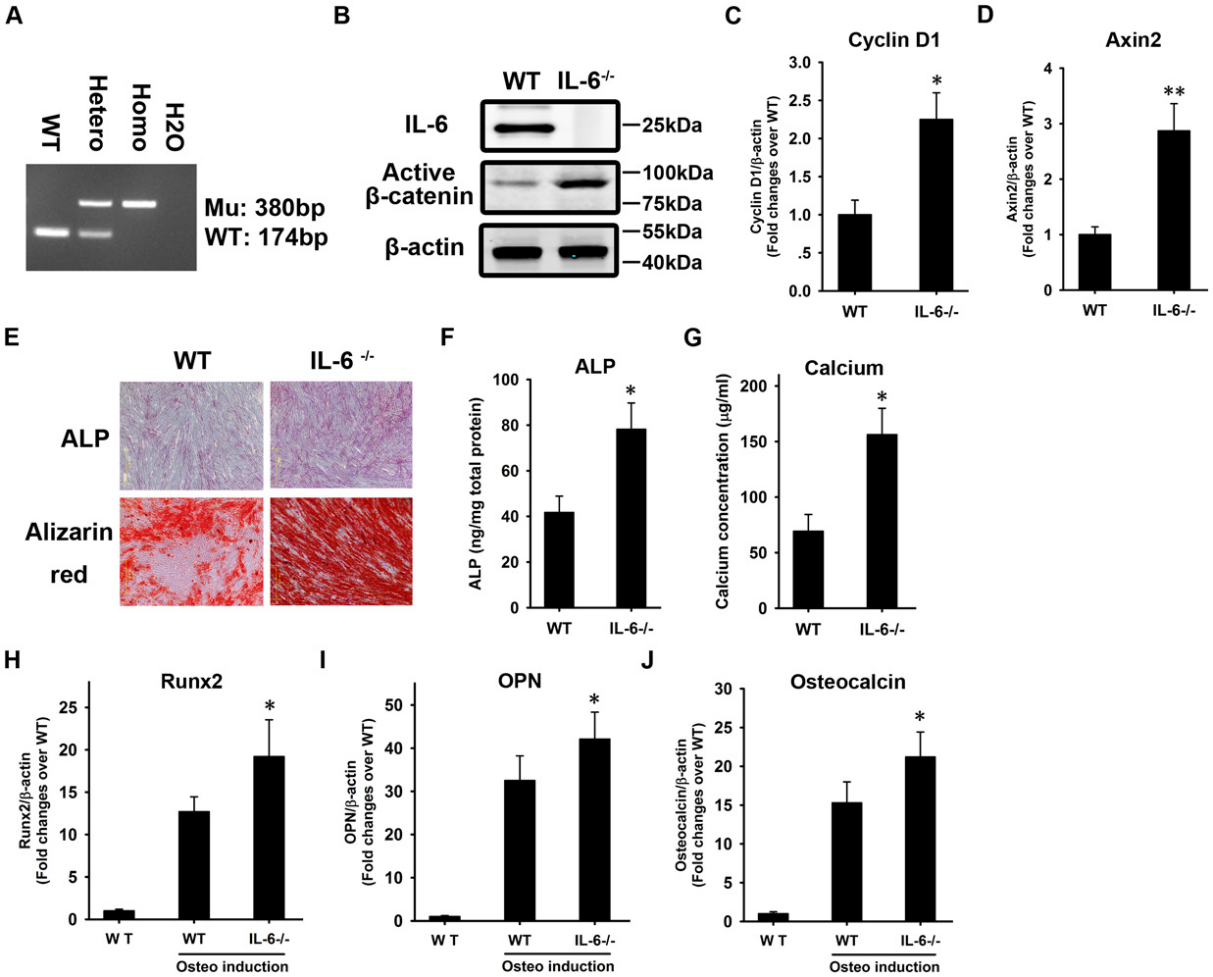
Knockout of IL-6 enhances the osteogenesis of BMSCs. IL-6^-/-^ BMSCs were isolated from the vertebral body of an IL-6^-/-^ mouse **(A)**. A wild-type (WT) mouse was used as a control. Total protein was harvested, and western blotting was carried out using antibodies against IL-6 and active β-catenin **(B)**. Total RNA was harvested for quantitative RT-PCR using primers for cyclin D1 (C) and Axin2 (D). The results are expressed as fold-changes relative to BMSCs from the wild-type mouse. WT and IL-6^-/-^ BMSCs were induced to undergo osteogenesis. Alkaline phosphatase (ALP) and Alizarin Red staining were performed at weeks 1 and 3, respectively **(E)**. The quantification of ALP **(F)** and measurement of calcium concentration **(G)** were performed. At week 2, total RNA was extracted and subjected to quantitative RT-PCR analysis using primers for Runx2 **(H)**, OPN **(I),** and OC **(J)**. β-actin was used as a control. The results are expressed as fold-changes relative to WT BMSCs without induction. The data are shown as the mean±SD. *: p<0.05 and **: p<0.01, IL-6^-/-^ *vs*. WT.

BMSCs were isolated from the vertebral body of an IL-6^-/-^ mouse (Fig 7A) that does not express IL-6 (Fig 7B). A significant activation of β-catenin was observed in the BMSCs from the IL-6^-/-^ mouse when compared with its activity in cells from a wild-type mouse (Fig 7B). In addition, there was a significant upregulation of the expression of the β-catenin target genes cyclin D1 (Fig 7C) and Axin2 (Fig 7D) in the BMSCs from the IL-6^-/-^ mouse compared with the expression in cells from a wild-type mouse.

BMSCs from IL-6^-/-^ and wild-type mice were induced to undergo osteogenesis *in vitro*. At week 1, ALP staining revealed that ALP activation was stronger in the IL-6^-/-^ BMSCs than in the wild-type BMSCs (Fig 7E). At week 3, Alizarin Red staining showed the same pattern as the ALP staining (Fig 7E). The quantification of ALP (Fig 7F) and calcium concentration (Fig 7G) supported the staining data. Total RNA was extracted at week 2 and subjected to quantitative RT-PCR to determine the expression of Runx2, OPN, and OC. The qRT-PCR revealed a more significant BMP2-induced activation of Runx2 (Fig 7H) and higher levels of OPN (Fig 7I) and OC (Fig 7J) mRNA in the IL-6^-/-^ BMSCs compared to the wild-type cells.

### *In vivo* administration of an IL-6 neutralizing antibody rescues the osteoporotic phenotype of mouse vertebrae

An IL-6 neutralizing antibody was used to treat mice in which placebo or prednisolone pellets had been implanted. The IL-6 neutralizing antibody enhanced the global and spinal BMD of mice treated with prednisolone (Table 3). The IL-6 neutralizing antibody enhanced serum osteocalcin levels in mice treated with prednisolone (Table 3). There was no significantly statistical difference in the level of serum CTX-I between with and without IL-6 neutralizing antibody treatment, although mild decrease was observed in response to IL-6 neutralizing antibody treatment compared with IgG control (Table 3).

**Table 3:** BMD and serum biochemical measurements in osteoporotic mice with and without IL-6 nAb administration. Data shown are the mean±SD. There are ten animals per group.

Parameters	Placebo	Prednisolone	Prednisolone+IgG	Prednisolone+IL-6 nAb
Global BMD (% change)	-2.2±1.1	-7.1±2.3*	-6.8±1.5*	-4.6±1.5*
Spinal BMD (% change)	-4.5±1.5	-9.9±2.3*	-8.1±1.3*	-5.8±1.9*
Osteocalcin (mg/L)	73.7±7.6	49.2±4.6**	53.2±6.6*	63.5±7.5*
CTX-I (ng/mL)	7.2±1.5	10.5±1.8*	11.8±2.2*	9.1±2.0

*P<0.05,

**P<0.01 Prednisolone *vs*. placebo, Prednisolone+IgG *vs*. Placebo and Prednisolone+IL-6 nAb *vs*. Prednisolone+IgG.

**Fig 8:**
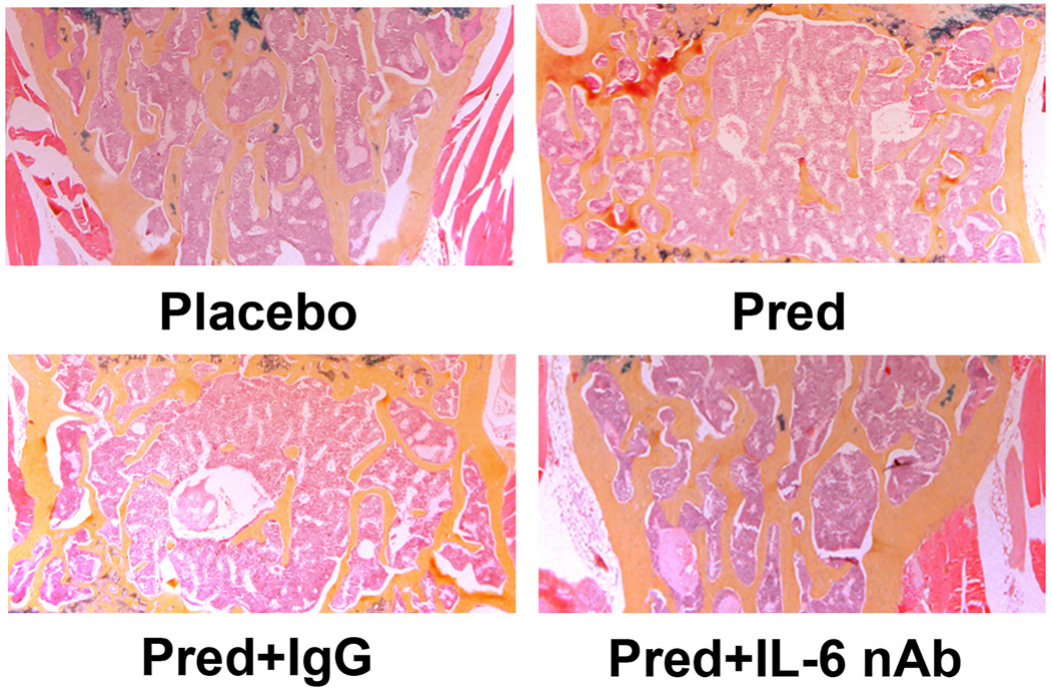
Cancellous bone staining of the vertebral body from an osteoporotic mouse treated with an IL-6 neutralizing antibody. An IL-6 neutralizing antibody was used to treat mice implanted with placebo or prednisolone pellets. IgG was used as a control. The vertebral bodies were cut into sections, and hematoxylin and orange G staining was performed.

The vertebral bodies of mice treated with or without the IL-6 neutralizing antibody were cut into sections. Staining revealed more cancellous bone within the vertebral bodies of mice receiving treatment with the IL-6 neutralizing antibody relative those receiving treatment with IgG (Fig 8A). Consistent with the staining data, bone histomorphometric analysis revealed a significant enhancement in the vertebral cancellous bone area, trabecular width, trabecular number and osteoblast surface (% bone surface) and a significant decline in trabecular spacing in mice treated with the IL-6 neutralizing antibody relative to those treated with IgG (Table 4). Relatively, IL-6 neutralizing antibody failed to lower osteoclast surface (% bone surface) in mice treated with prednisolone (Table 4). The data described above suggested that IL-6 neutralizing antibody rescued osteoporotic phenotype of GIO mouse mainly through enhancing bone formation but not suppressing bone resorption.

**Table 4:** Vertebral cancellous bone histomorphometry analysis in osteoporotic mice with and without IL-6 nAb administration. ObS: Osteoblast surface, OcS: Osteoclast surface, BS: bone surface. Data shown are the mean±SD. There are ten animals per group.

Histomorphometric parameters	Placebo	Prednisolone	Prednisolone+IgG	Prednisolone+IL-6 nAb
Bone area/tissue area (%)	19.7±4.3	5.9±1.1**	6.7±2.3*	12.9±3.4*
Trabecular thickness (μm)	35.6±4.6	22.4±3.5**	25.7±3.1	29.3±4.8*
Trabecular spacing (μm)	484±51	579±35*	597±26*	514±35*
Trabecular number (mm^-1^)	1.99±0.23	1.24±0.24*	1.06±0.19*	1.78±0.36*
ObS/BS (%)	35.72±5.97	27.38±3.95*	25.94±5.22*	33.25±4.62*
OcS/BS (%)	3.23±0.52	5.49±0.78*	5.74±0.66*	5.25±0.57

*P<0.05,

**P<0.01 Prednisolone *vs*. placebo, Prednisolone+IgG *vs*. Placebo and Prednisolone+IL-6 nAb *vs*. Prednisolone+IgG.

## Discussion

In review of osteoporosis research history, the researchers initially focused on bone resorption and osteoclast over-activation, and then on bone formation. Recently, BMSCs differentiation process has become a hot area with intense research interest. To elucidate the osteoporosis pathogenic mechanisms, it is necessary to understand the process of bone remodeling. Bone remodeling process initiates with the differentiation of hematopoietic precursors to osteoclasts. Because the bone resorption period of bone remodeling is relatively short and the period of osteoblastic replacement and bone formation is long, any increase in the rate of bone remodeling will lead to lower bone mass. Thus, multiple conditions in which an increase in osteoclastic resorption can result in skeletal fragility. However, high rates of bone resorption are not always equal to bone loss; for example, during the pubertal growth spurt. An inadequate bone formation in response to bone resorption during remodeling is an essential component of the pathogenesis of osteoporosis. Unlike menopause and senile osteoporosis in which bone resorption plays an essential role, a key feature of glucocorticoid-induced osteoporosis is defective bone formation, confirmed by the previous and current study [3]. Therefore, in the current study, our research interests were not mainly focused on the role of bone resorption, but bone formation, in glucocorticoid-induced osteoporosis, although we can not exclude the possibility of involvement of excessive bone resorption.

In the musculoskeletal system, osteoid synthesis and mineralization are carried out by osteoblasts originating from BMSCs. In osteoporosis, the BMSC osteogenic potential is defective [7]. Elucidating the molecular mechanisms underlying the defective BMSC osteogenesis will facilitate the prevention and treatment of osteoporosis.

Exploring the differences that exist between normal and osteoporotic BMSCs is of utmost importance. It was reported that osteoporotic BMSCs have increased adipogenesis, which is characterized by impaired leptin action [22]. Zhang *et al*. observed the attenuation of osteogenic potential and the enhancement of adipogenic potential in osteoporotic BMSCs in which epigenetic modifications in key transcriptional factors play important roles [6, 7]. Osteoporotic BMSCs differ from normal controls in that they have a lower growth rate and are refractory to the mitogenic effect of IGF-1, which is involved in the MAPK signaling pathway [23, 24]. The capacity of BMSCs derived from osteoporotic postmenopausal women to generate and maintain type I collagen-rich extracellular matrix is decreased, favoring the adipogenic differentiation of the cells [25]. Distinctive environmental bone marrow conditions appear to support the development and maintenance of the imbalance between bone resorption and bone formation in osteoporosis. These complex bone marrow conditions are reflected in the fluid surrounding bone marrow cells. In the current study, we compared 36 bone metabolism markers in normal and osteoporotic BMSCs using an antibody array. A significant upregulation of IL-6 levels was observed in osteoporotic BMSCs compared with normal controls.

Since its discovery 30 years ago, the list of IL-6 functions is still expanding. From a differentiation factor for macrophages to a hepatocyte stimulating factor and a B cell stimulatory factor, this cytokine is now revealed as a pleiotropic factor playing an important role in many biological events in the immune and the central nervous systems as well as bone [26–28]. Previous reports revealed the elevated production of IL-6 in RA, and indicated a causative role for IL-6 in disease activity [29]. Recent report suggested that Tocilizumab, a kind of monoclonal antibody against IL-6 receptor, positively modulates bone balance through suppressing bone resorption in RA patients [30, 31]. Soon, IL-6 appeared to be produced by stromal/osteoblastic cells and to enhance osteoclast formation by increasing interactions between osteoblasts and osteoclasts [32]. In contrast to bone resorption, the effects of IL-6 on bone formation remain an open question and some discrepant results are obtained. The elucidation of molecular mechanisms underlying the effects of IL-6 on bone formation is of utmost importance.

In the current study, we provided evidences that there was a negative relationship between IL-6 secretion and BMSC osteogenesis. Our findings are not unique. Many reports support the results of the current study. In IL-6 knock-out mice, there are microstructure abnormalities in cortical bones and delayed fracture healing were observed [33, 34], in spite of the grossly normal phenotype [35, 36]. Moreover, IL-6-overexpressed-transgenic mice appear to have osteopenia and defective ossification, in which the activity of mature osteoblasts is significantly decreased [37]. Kaneshiro *et al*. found that IL-6 negatively regulates osteoblast differentiation *in vitro* through the SHP2/MEK2 and SHP2/Akt2 pathways [38]. Poli *et al*. reported that IL-6-deficient mice are protected from bone loss caused by estrogen depletion [36]. Bian *et al*. reported that the IL-6 secreted by osteosarcoma cells inhibits BMSC osteogenic differentiation [39]. Some drugs could suppress bone loss via the inhibition of IL-6 expression [40–44]. Our findings differ from those of Huh *et al*. who found that IL-6 is produced by adipose-derived stromal cells and promotes osteogenesis [45]. The reason for this difference is unclear. However, it is possible that the different cell sources used in these two studies (bone marrow *vs*. adipose tissue) contributed to the conflicting results. In addition, a previous study by Bellido *et al*. demonstrated that IL-6-type cytokines promoted differentiation of committed osteoblastic cells toward a more mature phenotype and that this action is mediated primarily via the activation of the JAK/STAT pathway [46]. These findings differed from the results of current study. The reason for the discrepancy is not clear. We noticed that committed osteoblastic cell types, human (MG-63) and murine (MC3T3-E1) osteoblastic cell lines as well as primary murine calvaria cells, were employed in that study. And uncommitted bone marrow stromal cells were used in the current study. Maybe these dual effects depend on the differentiation stage of the osteoblast, that is to say, on precursor cells, IL-6-type cytokines would stimulate the first stages of differentiation but on more mature cells, they would prevent further stimulation.

To date, there are few reports describing the molecular mechanism by which IL-6 regulates BMSC osteogenesis. In a previous study, the authors found that IL-6 negatively regulates osteoblast differentiation *in vitro* through the SHP2/MEK2 and SHP2/Akt2 pathways [38]. In the current study, we provided evidence that β-catenin activation is inhibited in response to the upregulation of IL-6 in osteoporotic BMSCs. Considering the essential role that β-catenin plays in BMSC osteogenesis, we suggest that the wnt/β-catenin signaling pathway is involved in the IL-6-mediated regulation of BMSC osteogenesis. To the best of our knowledge, this is the first report to suggest that IL-6 negatively regulated BMSC osteogenesis by the inhibition of β-catenin. In a previous report, interesting data suggested that IL-6 accelerated calcification of human adipose tissue-derived mesenchymal stem cells. The mRNA transcription for receptor tyrosine kinase-like orphan receptor 2 (ROR2), involved in the non-canonical wingless-type (WNT) MMTV integration site pathway, was increased, while β-catenin expression, an essential factor in the canonical WNT signalling pathway for osteoblast differentiation, did not change [47]. Together with the data from current study, we speculate that the discrepancy of IL-6 effect on osteogenic differentiation is likely to be resulted from the canonical and non-canonical Wnt signaling pathway, that is to say, if canonical Wnt signaling is inhibited by IL-6, osteogenic differentiation will be inhibited; if non-canonical Wnt signaling pathway is induced by IL-6, osteogenic differentiation will be promoted.

The most important data in the current study came from the *in vivo* experiments in which an IL-6 neutralizing antibody was administered to osteoporotic mice. Treatment with the neutralizing antibody rescued the osteoporotic phenotype of the vertebral body, including BMD, serum osteocalcin concentration, and bone histomorphometric parameters. Many reports have confirmed the role of IL-6 in the pathogenesis of various diseases [48]. In B cell malignancies, IL-6 promoted the growth and inhibited the apoptosis of multiple myeloma cells [49–51]. In addition, IL-6 is regarded as a key factor of postmenopausal osteoporosis due to its ability to activate osteoclast and induce bone resoption [52]. IL-6 is also involved in the pathophysiology of rheumatoid arthritis and multiple sclerosis. Based on these data, IL-6 blockage is a promising way to treat several pathological conditions with IL-6 excessive production. However, the administration of IL-6 antibody does not neutralize IL-6 activity efficiently *in vivo*, which might be attributed to the stabilization of IL-6 in monomeric complexes and then the accumulation of the cytokine in the circulation. Our data suggest that the *in vivo* administration of an IL-6 neutralizing antibody is effective in rescuing the osteoporotic phenotype of the vertebral body, at least in mice.

In conclusion, our study revealed that IL-6 contributes to the defective osteogenesis of BMSCs in the vertebral body of the osteoporotic mouse and that the *in vivo* administration of an IL-6 neutralizing antibody can rescue this phenotype. Our study provides deeper insight into the pathophysiology of osteoporosis and identifies IL-6 as a promising target for the treatment of osteoporosis.

## Supporting Information

S1 Table Primer sequences used in RT-PCR. (DOC)

S1 Fig The expression profiles of G-CSF, leptin, oncostatin M and IL-11 in BMSCs from placebo and prednisolone group. (DOC)

## References

[1] Cushing H. The basophil adenomas of the pituitary body and their clinical manifestations (pituitary basophilism). 1932. Obesity research. 1994;2(5):486–508. . 16353601 10.1002/j.1550-8528.1994.tb00097.x

[2] Reid IR. Pathogenesis and treatment of steroid osteoporosis. Clinical endocrinology. 1989;30(1):83–103. . 2673589 10.1111/j.1365-2265.1989.tb03730.x

[3] Weinstein RS, Jilka RL, Parfitt AM, Manolagas SC. Inhibition of osteoblastogenesis and promotion of apoptosis of osteoblasts and osteocytes by glucocorticoids. Potential mechanisms of their deleterious effects on bone. The Journal of clinical investigation. 1998;102(2):274–82. 10.1172/JCI2799 9664068 PMC508885

[4] Kendler DL, Bauer DC, Davison KS, Dian L, Hanley DA, Harris ST, et al Vertebral fractures: clinical importance and management. The American journal of medicine. 2015 10.1016/j.amjmed.2015.09.020 . 26524708

[5] Dempster DW. Bone histomorphometry in glucocorticoid-induced osteoporosis. Journal of bone and mineral research: the official journal of the American Society for Bone and Mineral Research. 1989;4(2):137–41. 10.1002/jbmr.5650040202 . 2658477

[6] Zhang Y, Ma C, Liu X, Wu Z, Yan P, Ma N, et al Epigenetic landscape in PPARgamma2 in the enhancement of adipogenesis of mouse osteoporotic bone marrow stromal cell. Biochimica et biophysica acta. 2015;1852(11):2504–16. 10.1016/j.bbadis.2015.08.020 . 26319419

[7] Zhang YX, Sun HL, Liang H, Li K, Fan QM, Zhao QH. Dynamic and distinct histone modifications of osteogenic genes during osteogenic differentiation. Journal of biochemistry. 2015;158(6):445–57. 10.1093/jb/mvv059 . 26078467

[8] Raisz LG. Pathogenesis of osteoporosis: concepts, conflicts, and prospects. The Journal of clinical investigation. 2005;115(12):3318–25. 10.1172/JCI27071 16322775 PMC1297264

[9] Sambrook P, Cooper C. Osteoporosis. Lancet. 2006;367(9527):2010–8. 10.1016/S0140-6736(06)68891-0 . 16782492

[10] Nuttall ME, Gimble JM. Controlling the balance between osteoblastogenesis and adipogenesis and the consequent therapeutic implications. Current opinion in pharmacology. 2004;4(3):290–4. 10.1016/j.coph.2004.03.002 . 15140422

[11] Tontonoz P, Hu E, Graves RA, Budavari AI, Spiegelman BM. mPPAR gamma 2: tissue-specific regulator of an adipocyte enhancer. Genes & development. 1994;8(10):1224–34. . 7926726 10.1101/gad.8.10.1224

[12] Khosla S, Atkinson EJ, Dunstan CR, O'Fallon WM. Effect of estrogen versus testosterone on circulating osteoprotegerin and other cytokine levels in normal elderly men. The Journal of clinical endocrinology and metabolism. 2002;87(4):1550–4. 10.1210/jcem.87.4.8397 . 11932280

[13] Koshihara Y, Suematsu A, Feng D, Okawara R, Ishibashi H, Yamamoto S. Osteoclastogenic potential of bone marrow cells increases with age in elderly women with fracture. Mechanisms of ageing and development. 2002;123(10):1321–31. . 12297335 10.1016/s0047-6374(02)00071-4

[14] Ferrari SL, Ahn-Luong L, Garnero P, Humphries SE, Greenspan SL. Two promoter polymorphisms regulating interleukin-6 gene expression are associated with circulating levels of C-reactive protein and markers of bone resorption in postmenopausal women. The Journal of clinical endocrinology and metabolism. 2003;88(1):255–9. 10.1210/jc.2002-020092 . 12519862

[15] Scheidt-Nave C, Bismar H, Leidig-Bruckner G, Woitge H, Seibel MJ, Ziegler R, et al Serum interleukin 6 is a major predictor of bone loss in women specific to the first decade past menopause. The Journal of clinical endocrinology and metabolism. 2001;86(5):2032–42. 10.1210/jcem.86.5.7445 . 11344203

[16] Barbour KE, Boudreau R, Danielson ME, Youk AO, Wactawski-Wende J, Greep NC, et al Inflammatory markers and the risk of hip fracture: the Women's Health Initiative. Journal of bone and mineral research: the official journal of the American Society for Bone and Mineral Research. 2012;27(5):1167–76. 10.1002/jbmr.1559 22392817 PMC3361578

[17] Ding C, Parameswaran V, Udayan R, Burgess J, Jones G. Circulating levels of inflammatory markers predict change in bone mineral density and resorption in older adults: a longitudinal study. The Journal of clinical endocrinology and metabolism. 2008;93(5):1952–8. 10.1210/jc.2007-2325 . 18285417

[18] Parfitt AM, Drezner MK, Glorieux FH, Kanis JA, Malluche H, Meunier PJ, et al Bone histomorphometry: standardization of nomenclature, symbols, and units. Report of the ASBMR Histomorphometry Nomenclature Committee. Journal of bone and mineral research: the official journal of the American Society for Bone and Mineral Research. 1987;2(6):595–610. 10.1002/jbmr.5650020617 . 3455637

[19] Falla N, Van V, Bierkens J, Borremans B, Schoeters G, Van Gorp U. Characterization of a 5-fluorouracil-enriched osteoprogenitor population of the murine bone marrow. Blood. 1993;82(12):3580–91. . 8260697

[20] Yadav VK, Ducy P. Lrp5 and bone formation: A serotonin-dependent pathway. Annals of the New York Academy of Sciences. 2010;1192:103–9. 10.1111/j.1749-6632.2009.05312.x . 20392224

[21] De Vos J, Jourdan M, Tarte K, Jasmin C, Klein B. JAK2 tyrosine kinase inhibitor tyrphostin AG490 downregulates the mitogen-activated protein kinase (MAPK) and signal transducer and activator of transcription (STAT) pathways and induces apoptosis in myeloma cells. British journal of haematology. 2000;109(4):823–8. . 10929036 10.1046/j.1365-2141.2000.02127.x

[22] Astudillo P, Rios S, Pastenes L, Pino AM, Rodriguez JP. Increased adipogenesis of osteoporotic human-mesenchymal stem cells (MSCs) characterizes by impaired leptin action. Journal of cellular biochemistry. 2008;103(4):1054–65. 10.1002/jcb.21516 . 17973271

[23] Rodriguez JP, Garat S, Gajardo H, Pino AM, Seitz G. Abnormal osteogenesis in osteoporotic patients is reflected by altered mesenchymal stem cells dynamics. Journal of cellular biochemistry. 1999;75(3):414–23. . 10536365 10.1002/(sici)1097-4644(19991201)75:3<414::aid-jcb7>3.3.co;2-3

[24] Rodriguez JP, Rios S, Fernandez M, Santibanez JF. Differential activation of ERK1,2 MAP kinase signaling pathway in mesenchymal stem cell from control and osteoporotic postmenopausal women. Journal of cellular biochemistry. 2004;92(4):745–54. 10.1002/jcb.20119 . 15211572

[25] Rodriguez JP, Montecinos L, Rios S, Reyes P, Martinez J. Mesenchymal stem cells from osteoporotic patients produce a type I collagen-deficient extracellular matrix favoring adipogenic differentiation. Journal of cellular biochemistry. 2000;79(4):557–65. . 10996846 10.1002/1097-4644(20001215)79:4<557::aid-jcb40>3.0.co;2-h

[26] Heymann D, Rousselle AV. gp130 Cytokine family and bone cells. Cytokine. 2000;12(10):1455–68. 10.1006/cyto.2000.0747 . 11023660

[27] Liu XH, Kirschenbaum A, Yao S, Levine AC. The role of the interleukin-6/gp130 signaling pathway in bone metabolism. Vitamins and hormones. 2006;74:341–55. 10.1016/S0083-6729(06)74014-6 . 17027522

[28] Wong PK, Campbell IK, Egan PJ, Ernst M, Wicks IP. The role of the interleukin-6 family of cytokines in inflammatory arthritis and bone turnover. Arthritis and rheumatism. 2003;48(5):1177–89. 10.1002/art.10943 . 12746890

[29] Houssiau FA, Devogelaer JP, Van Damme J, de Deuxchaisnes CN, Van Snick J. Interleukin-6 in synovial fluid and serum of patients with rheumatoid arthritis and other inflammatory arthritides. Arthritis and rheumatism. 1988;31(6):784–8. . 3260102 10.1002/art.1780310614

[30] Karsdal MA, Schett G, Emery P, Harari O, Byrjalsen I, Kenwright A, et al IL-6 receptor inhibition positively modulates bone balance in rheumatoid arthritis patients with an inadequate response to anti-tumor necrosis factor therapy: biochemical marker analysis of bone metabolism in the tocilizumab RADIATE study (NCT00106522). Seminars in arthritis and rheumatism. 2012;42(2):131–9. 10.1016/j.semarthrit.2012.01.004 . 22397953

[31] Terpos E, Fragiadaki K, Konsta M, Bratengeier C, Papatheodorou A, Sfikakis PP. Early effects of IL-6 receptor inhibition on bone homeostasis: a pilot study in women with rheumatoid arthritis. Clinical and experimental rheumatology. 2011;29(6):921–5. . 22032557

[32] Blanchard F, Duplomb L, Baud'huin M, Brounais B. The dual role of IL-6-type cytokines on bone remodeling and bone tumors. Cytokine & growth factor reviews. 2009;20(1):19–28. 10.1016/j.cytogfr.2008.11.004 . 19038573

[33] Sims NA, Jenkins BJ, Nakamura A, Quinn JM, Li R, Gillespie MT, et al Interleukin-11 receptor signaling is required for normal bone remodeling. Journal of bone and mineral research: the official journal of the American Society for Bone and Mineral Research. 2005;20(7):1093–102. 10.1359/JBMR.050209 . 15940362

[34] Yang X, Ricciardi BF, Hernandez-Soria A, Shi Y, Pleshko Camacho N, Bostrom MP. Callus mineralization and maturation are delayed during fracture healing in interleukin-6 knockout mice. Bone. 2007;41(6):928–36. 10.1016/j.bone.2007.07.022 17921078 PMC2673922

[35] Kopf M, Baumann H, Freer G, Freudenberg M, Lamers M, Kishimoto T, et al Impaired immune and acute-phase responses in interleukin-6-deficient mice. Nature. 1994;368(6469):339–42. 10.1038/368339a0 . 8127368

[36] Poli V, Balena R, Fattori E, Markatos A, Yamamoto M, Tanaka H, et al Interleukin-6 deficient mice are protected from bone loss caused by estrogen depletion. The EMBO journal. 1994;13(5):1189–96. 8131749 10.1002/j.1460-2075.1994.tb06368.x PMC394928

[37] De Benedetti F, Rucci N, Del Fattore A, Peruzzi B, Paro R, Longo M, et al Impaired skeletal development in interleukin-6-transgenic mice: a model for the impact of chronic inflammation on the growing skeletal system. Arthritis and rheumatism. 2006;54(11):3551–63. 10.1002/art.22175 . 17075861

[38] Kaneshiro S, Ebina K, Shi K, Higuchi C, Hirao M, Okamoto M, et al IL-6 negatively regulates osteoblast differentiation through the SHP2/MEK2 and SHP2/Akt2 pathways in vitro. Journal of bone and mineral metabolism. 2014;32(4):378–92. 10.1007/s00774-013-0514-1 . 24122251

[39] Bian ZY, Fan QM, Li G, Xu WT, Tang TT. Human mesenchymal stem cells promote growth of osteosarcoma: involvement of interleukin-6 in the interaction between human mesenchymal stem cells and Saos-2. Cancer science. 2010;101(12):2554–60. 10.1111/j.1349-7006.2010.01731.x . 20874851 PMC11159660

[40] Park JH, Park KH, Cho S, Choi YS, Seo SK, Lee BS, et al Concomitant increase in muscle strength and bone mineral density with decreasing IL-6 levels after combination therapy with alendronate and calcitriol in postmenopausal women. Menopause. 2013;20(7):747–53. 10.1097/GME.0b013e31827cabca . 23511701

[41] Hattori T, Fei W, Kizawa T, Nishida S, Yoshikawa H, Kishida Y. The fixed herbal drug composition "Saikokaryukotsuboreito" prevents bone loss with an association of serum IL-6 reductions in ovariectomized mice model. Phytomedicine: international journal of phytotherapy and phytopharmacology. 2010;17(3–4):170–7. 10.1016/j.phymed.2009.12.004 . 20097049

[42] Olmos JM, De Vega T, Perera L, Riancho JA, Amado JA, Gonzalez-Macias J. Etidronate inhibits the production of IL-6 by osteoblast-like cells. Methods and findings in experimental and clinical pharmacology. 1999;21(8):519–22. . 10599049 10.1358/mf.1999.21.8.794832

[43] Romer P, Desaga B, Proff P, Faltermeier A, Reicheneder C. Strontium promotes cell proliferation and suppresses IL-6 expression in human PDL cells. Annals of anatomy = Anatomischer Anzeiger: official organ of the Anatomische Gesellschaft. 2012;194(2):208–11. 10.1016/j.aanat.2011.09.008 . 22051238

[44] Yamaguchi K, Yada M, Tsuji T, Hatanaka Y, Goda K, Kobori T. 4-Phenylthiazole derivatives inhibit IL-6 secretion in osteoblastic cells and suppress bone weight loss in ovariectomized mice. Bioorganic & medicinal chemistry letters. 1999;9(7):957–60. . 10230619 10.1016/s0960-894x(99)00122-5

[45] Huh JE, Lee SY. IL-6 is produced by adipose-derived stromal cells and promotes osteogenesis. Biochimica et biophysica acta. 2013;1833(12):2608–16. 10.1016/j.bbamcr.2013.06.025 . 23830919

[46] Bellido T, Borba VZ, Roberson P, Manolagas SC. Activation of the Janus kinase/STAT (signal transducer and activator of transcription) signal transduction pathway by interleukin-6-type cytokines promotes osteoblast differentiation. Endocrinology. 1997;138(9):3666–76. 10.1210/endo.138.9.5364 . 9275051

[47] Fukuyo S, Yamaoka K, Sonomoto K, Oshita K, Okada Y, Saito K, et al IL-6-accelerated calcification by induction of ROR2 in human adipose tissue-derived mesenchymal stem cells is STAT3 dependent. Rheumatology. 2014;53(7):1282–90. 10.1093/rheumatology/ket496 . 24599911

[48] De Benedetti F, Pignatti P, Vivarelli M, Meazza C, Ciliberto G, Savino R, et al In vivo neutralization of human IL-6 (hIL-6) achieved by immunization of hIL-6-transgenic mice with a hIL-6 receptor antagonist. Journal of immunology. 2001;166(7):4334–40. . 11254686 10.4049/jimmunol.166.7.4334

[49] Hallek M, Bergsagel PL, Anderson KC. Multiple myeloma: increasing evidence for a multistep transformation process. Blood. 1998;91(1):3–21. 9414264 PMC3901996

[50] Hilbert DM, Kopf M, Mock BA, Kohler G, Rudikoff S. Interleukin 6 is essential for in vivo development of B lineage neoplasms. The Journal of experimental medicine. 1995;182(1):243–8. 7790819 10.1084/jem.182.1.243 PMC2192088

[51] Lattanzio G, Libert C, Aquilina M, Cappelletti M, Ciliberto G, Musiani P, et al Defective development of pristane-oil-induced plasmacytomas in interleukin-6-deficient BALB/c mice. The American journal of pathology. 1997;151(3):689–96. 9284817 PMC1857831

[52] Tamura T, Udagawa N, Takahashi N, Miyaura C, Tanaka S, Yamada Y, et al Soluble interleukin-6 receptor triggers osteoclast formation by interleukin 6. Proceedings of the National Academy of Sciences of the United States of America. 1993;90(24):11924–8. 8265649 10.1073/pnas.90.24.11924 PMC48097

