# Supplementary material for: IL-6 Contributes to the Defective Osteogenesis of Bone Marrow Stromal Cells from the Vertebral Body of the Glucocorticoid-Induced Osteoporotic Mouse
Source: PLoS One. 2016 Apr 29;11(4):e0154677. doi: 10.1371/journal.pone.0154677 (PMC4851291; doi:10.1371/journal.pone.0154677)
Supplement: S1 Table — (DOC) [file pone.0154677.s001.doc]

**S1 table**: Primer sequences used in RT-PCR

| **Gene** | **Primer sequences (5’-3’)** | **Product length (bp)** |
| --- | --- | --- |
| RANKL | CGAGCGCAGATGGATCCTAA (F) | 247 |
|  | CCACATCCAACCATGAGCCT (R) |  |
| OPG | ACAGTTTGCCTGGGACCAAA (F) | 160 |
|  | TCACAGAGGTCAATGTCTTGGA (R) |  |
| Runx2 | TTCAACGATCTGAGATTTGTGGG (F) | 221 |
|  | GGATGAGGAATGCGCCCTA (R) |  |
| OPN | CTGGCAGCTCAGAGGAGAAG (F) | 106 |
|  | TTCTGTGGCGCAAGGAGATT (R) |  |
| OC | GCTACCTTGGAGCCTCAGTC (F) | 71 |
|  | AGGGTTAAGCTCACACTGCT (R) |  |
| IL-6 | GCCTTCTTGGGACTGATGCT (F) | 475 |
|  | TGGAAATTGGGGTAGGAAGGAC (R) |  |
| Cyclin D1 | TCAAGTGTGACCCGGACTG (F) | 175 |
|  | ATGTCCACATCTCGCACGTC (R) |  |
| Axin2 | AGGTCCTGGCAACTCAGTAAC (F) | 164 |
|  | TCTCTTAAGTCAGCAGGGGC (R) |  |
| Dkk1 | TCTCTATGAGGGCGGGAACA (F) | 156 |
|  | TTTCGGCAAGCCAGACAGAT (R) |  |
| β-actin | GGCTGTATTCCCCTCCATCG (F) | 154 |
|  | CCAGTTGGTAACAATGCCATGT (R) |  |
