# Supplementary material for: IL-6 Contributes to the Defective Osteogenesis of Bone Marrow Stromal Cells from the Vertebral Body of the Glucocorticoid-Induced Osteoporotic Mouse
Source: PLoS One. 2016 Apr 29;11(4):e0154677. doi: 10.1371/journal.pone.0154677 (PMC4851291; doi:10.1371/journal.pone.0154677)
Supplement: S1 Fig — (DOC) [file pone.0154677.s002.doc]

**
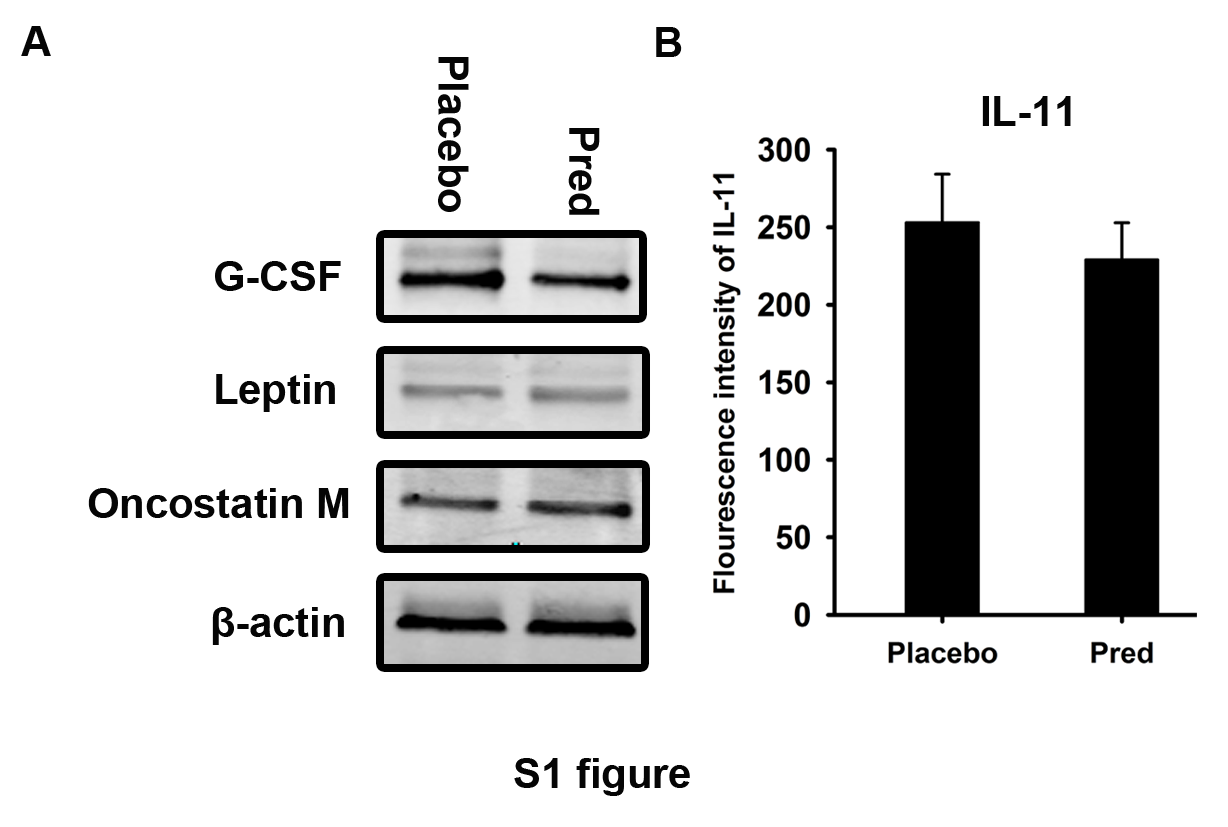
**

**S1 figure. The expression profiles of G-CSF, leptin, oncostatin M and IL-11 in BMSCs from placebo and prednisolone group.** BMSCs were isolated from the vertebral bodies of mice treated with the placebo or prednisolone pellets. Total proteins were harvested for western blot **(A)** using G-CSF, leptin and oncostatin M antibodies and antibody array **(B)** in which IL-11 expression level was represented as fluorescence intensity. β-actin was used as internal control for western blot. The data are shown as the mean±SD.
